# Pathology, Practical Medicine, and Therapeutics

**Published:** 1837-10

**Authors:** 


### PATHOLOGY, PRACTICAL MEDICINE, AND THERAPEUTICS.

### *On Pneumonia of the Old.* By MM. HOURMANN and DECHAMBRE.

[This is the continuation of the excellent Memoir by the same authors, the preceding parts of which we published in former Numbers. See Vol. I. p. 233, and Vol. II. p. 543.]

*Etiology and Symptomatology of Pneumonia.* The following facts are founded on 109 cases, carefully examined from day to day: of these, 33 were cured; 67 died, and were examined after death; 9 died, with well-marked symptoms of pneumonia, but no dissection was made.

### I. *Causes of Pneumonia in the Old.*

*Predisposing Causes.* The most active predisposing cause is habitual bronchorrhœa and permanent congestion, the frequency of which has been previously mentioned. Less direct causes are, the rigidity of the whole mechanical apparatus of respiration, which impedes the passage of air, the expectoration of mucus, and the pulmonary circulation; also organic affections of the heart and great vessels, so common in old age; distention of the abdomen from various causes, and particularly from gas accumulated in the intestines, and pressing up the diaphragm; debility from age or diseases; acute bronchitis, which occasionally supervenes on the chronic and chronic pneumonia. Finally, inflammation of one lung predisposes the other to inflammation, so that double pneumonia is very common.

*Occasional Causes.* 1. *Cold.* It is constantly remarked at the Salpetrière, that, as the thermometer descends, the number of patients increases, and five-sixths are of pneumonia. One hundred and fifty-six patients were thus distributed.

| | | | | |
|---|---|---|---|---|
| In January . . | 17 cases | .. | 6 deaths. |
| February . . | 19 — | .. | 14 — |
| March . . | 31 — | .. | 20 — |
| April . . . | 20 — | .. | 9 — |
| May . . . | 11 — | .. | 3 — |
| June . . . | 6 — | .. | 4 — |
| July . . . | 3 — | .. | 2 — |
| August . . | 1 — | .. | 1 — |
| September . | 1 — | .. | 0 — |
| October . . | 5 — | .. | 4 — |
| November . | 19 — | .. | 15 — |
| December . | 23 — | .. | 10 — |
| | 156 | | 88 |

2. *Variations in Temperature.* Sudden changes of temperature are frequent causes. The preceding table shows this, besides proving that pneumonia is more frequent in the cold months; for the maximum of frequency is neither in the coldest month, as January, nor in the hottest, as July; but in March, and the first fortnight of April, when sudden changes of temperature are common.

3. *Winds.* Dry and strong winds favour the development of pneumonia, particularly the north-east.

4. *Lying in one position for a length of time* produces congestion of the most dependent parts of the lungs, and thus is a cause of pneumonia. Hence pneumonia, as was formerly stated, most frequently occupies those parts of the lungs which are the lowest, when the patient lies on the back: pneumonia of the right lower lobe is most frequent, and the patients generally lie inclining towards the right side, whilst pneumonia of the left lower lobe is much more rare than of the left upper lobe. Again, in asthmatics who die in a state of orthopnœa, the bases of the lungs are often found to be greatly congested, or even hepatized. This, however, requires the concurrence of other causes, and its effects are much favoured by organic predispositions, particularly plethora: thus, prolonged dorsal decubitus is much less injurious to the thin cachectic old woman than to the fat and sanguineous old woman of the first class, which was described in a former article.*

*Mode of Attack.* This is twofold: either sudden, and with the common symptoms with which it attacks adults; or slowly, secretly, and without symptoms.

1. In the first form, it sometimes begins with a sudden rigor followed by pain in the side, or more frequently by pain in the side only, or this is followed by horripilations. When there is a rigor, it is followed by heat and slight perspiration, and but very rarely by sweating. At the Salpetrière, in March and April, pneumonia commences with peculiar severity.

2. In the second form, it neither begins with rigor nor with sharp pain in the

* British and Foreign Medical Review. No. IV. P. 546.

side; frequently only general uneasiness, weakness, increased frequency or irregularity of respiration, slight hacking cough, and heat of skin; or even nothing but general uneasiness and weakness. In some cases, an habitual cough ceased at the time when pneumonia commenced. Finally, sometimes the patients make no complaint: they rise, make their beds, eat as usual; then, feeling a little fatigue, they recline on their beds and expire. On examination, a considerable portion of the lungs has been found in a state of suppuration.

In those cases where there were organic diseases, such as diseases of the heart and brain, which might act as predisposing causes of pneumonia, the commencement of the attack was almost constantly latent; whilst, where there was no such predisposition, the symptoms were acute in rather more than half the cases.

II. *Symptoms of Pneumonia in the Old.*

(1.) *Rational Signs. Pain.* In the majority of cases, the pain is undetermined, sometimes in the whole side of the thorax which is affected, sometimes in the whole chest, and particularly its anterior part. This pain is, although diffuse, occasionally increased by the slightest pressure. *Dyspnœa.* The patients often complain of no difficulty of respiration, and it may be in such cases that the movements of the chest are in no degree modified. At others, however, they are very irregular, both in force and frequency, and the countenance is marked by great anxiety. The absence, more or less complete, of dyspnœa, is particularly observed in pneumonia of the lower lobe; but, when the inflammation is in the upper lobe, the dyspnœa is frequently a true asphyxia. There are cases, however, of latent pneumonia of the upper lobe. The breath frequently smells like pus, in the third degree. *Cough* generally exists, but it is sometimes so feeble as to excite no attention. The characteristic *Expectoration* observed in the pneumonia of the adult is very rarely seen in the old. It is frequently entirely absent, and often, when it does exist, it is but for a very short time. Its appearances are very variable. Sanguinolent expectoration is most frequent in those cases the commencement of which is acute. The mixture of blood, external to the body, with the secretions produced in chronic bronchitis, produces a fluid which resembles exactly the sanguinolent expectoration which sometimes accompanies the pneumonia of the old.

(2.) *Physical Signs.—Auscultation.—First degree.* Generally, the respiratory murmur is feeble at the point where the pneumonia is developed; sometimes, however, it is strong, and soon becomes bronchial. When strong, it is always accompanied by sonorous rhonchi. The crepitant rhonchus, as heard in the adult, is very rare. The bubbles which constitute it are almost always larger and more moist; the cells of the pulmonary parenchyma being enlarged in the aged, and the air passing through a mucous fluid. But most frequently the first degree of pneumonia gives rise only to a mucous rhonchus, of various extent. This is sometimes a true *gargouillement,* owing to the quantity of mucus which is secreted, and which is increased by the inflammation and the difficulty of expectoration. But it is chiefly in the lungs of the second and third type that this form of rhonchus is observed. Owing to the size of the bronchial tubes, the "souffle tubaire" is very frequent; audible at the root of the diseased lung, alternating momentarily with the natural respiration.

*Second degree.* The "souffle tubaire" almost always exists; is frequently accompanied by crackling or gurgling; and the combination of these sounds is like those which are produced by a cavity in the pulmonary parenchyma. The resonance of the voice is not so constantly associated with the bronchial respiration as in the adult, and its character is rather that of œgophony than of bronchophony.

*Third degree.* The signs of this degree are commonly confounded with those of the second; but occasionally the resonance of the voice and the bronchial respiration diminish in proportion to the progress of the pneumonia. The mucous rhonchus then often becomes more and more abundant, and its gurgling may be heard in all parts of the chest.

But pneumonia in old people has passed through all its stages without giving rise to any rhonchus. The various sounds which attend pneumonia in the old, and which extend far beyond the parts in which they are generated, show how the disease may be masked and remain latent in its most advanced stages.

*Percussion.* Before hepatization, the sound on percussion is more modified than in the adult; but this modification is relative, and we should consider a sound as obscure in the aged which would be clear in the adult. Hence, also, hepatization does not produce an absolute dulness, and particularly when the disease is in the anterior and upper part of the chest. The dulness is always more marked behind. Of course, in practising percussion, what has already been said of the varying sonorousness of the different parts of the thorax in the old must always be borne in mind.

(3.) *General Symptoms.* We have already seen that pneumonia may go on to a fatal termination, without having given rise to any manifest general symptoms. It is rare for pneumonia to run through all its stages without some delirium, continuous or interrupted, tranquil or agitated, and always increasing towards the night. Sometimes the mental faculties appear to be simply weakened. But it is important not to confound this state with adynamia, properly so called; for the former may be associated with complete integrity of muscular action and decided febrile reaction: this delirious state is frequent in other diseases of old people, but not in so early a stage as in pneumonia. There may be obstinate constipation or diarrhœa; the latter disposed to establish itself permanently after the use of purgatives. The tongue offers very various characters. Force and fulness of pulse, heat and moisture of skin, so generally connected with the pneumonia of the adult, are most rarely united with that of the aged. The pulse of the aged cannot be considered of morbid frequency, if it be less than ninety. This frequency is often not manifested until several days subsequent to the invasion of the disease, and in some cases not until its last period. The force of the pulse is a symptom not so often observed as its frequency.

The pulse is frequently irregular, and this happens more often where there is simple engorgement than where there is decided inflammation. The state of the skin, as to warmth and moisture, is subject to great variety. The fever which is observed to accompany the pneumonia of the aged may be classed as inflammatory, adynamic, and ataxic. The inflammatory fever is very rare. The adynamic fever is more common, and it presents two varieties. In one there is nothing special in the commencement of the disease: it may be acute or latent, the latter being more frequent. But, as the pneumonia advances, the prostration becomes extreme. The other variety is doubtless the adynamic fever of Pinel; he, however, having overlooked the condition of the lungs in his account of the disease. One of the most remarkable characteristics of the pneumonia of the aged is *the state of the blood.* It is only in the minority of cases that it presents the inflammatory crust; and this is most evident when the inflammation is acute. The clot is soft, and black or greenish; and sometimes the blood does not coagulate, but remains like molasses. The *progress* of the pneumonia of the old is very rapid.

With regard to the discovery of the existence of pneumonia in the aged, it should be always borne in mind that, whatever may be the complaint under which an elderly person may labour, a daily examination of the chest should be instituted. Symptoms of general discomfort (*malaise,*) should direct the physician's attention to the condition of the lungs. The *termination* of pneumonia in the aged in *complete* recovery is rare. Old women continue to cough, without complaining of any indisposition; and the signs discovered by auscultation show that the healthy condition is not quite restored. There is great disposition to relapse, and, on examination after death, are found spots of chronic induration of the lung, together with red hepatization of recent date. These indurated spots are sometimes very numerous.

The *prognosis* is, of course, highly unfavorable.

The cases observed by our authors, and the circumstances under which they were observed, prevent their offering any very decisive suggestions as to the *treatment* of the pneumonia of the aged. They appear to have followed the usual routine in similar cases; always bearing in mind the activity and character of the local disease in relation to the age of those who were the subjects of their treatment.

*Archives générales de Médecine.* Tome xii. *Sept. and Oct.* 1836.

---

### *On St. Vitus's Dance.* By DR. STIEBEL.

NEARLY one hundred cases of this disease have come under the notice of Dr. Stiebel; and in not one of these, he says, was wanting the evidence of an irritation of the spinal nerves. Few of the patients, during the course of the disease, have not had pain in some one of the vertebræ, and all, either in consequence of the treatment employed or spontaneously, have recovered. Dr. Stiebel believes that the cause of this disease is an irritation of the motor nerves of the spinal marrow or of the medulla oblongata, depending on inflammation or turgescence. Chorea almost always originates during the development of the spine and of the spinal marrow; generally between the seventh and seventeenth years, but occasionally at later periods of life.

The following appears to be the anatomical explanation of the disease:—The spinal marrow, and the origins of its nerves, lie within a bony cavity. If, during their development, there occurs a want of relation between the bones and the nervous system, so that the cavity does not correspond to the increasing marrow, the nervous origins become subject to an irritation as of a foreign body. This disproportion may be the effect of swelling of the spine, without previous change in the nerves, as well as of turgescence of the membranes of the nerves and the nerves themselves, the spine remaining unaltered; but the first is of most frequent occurrence. The spasms generally cease during sleep, although the irritation continues. The chorea may be limited to one side of the body, or to parts of less extent, according to the situation of the irritation. It is liable also to change its place, to become general after having been but partial, and *vice versâ.* It may also alternate with paralysis, which is as little dangerous as the original disease. The change of locality of the disease is not sudden, as in hysteric spasms, but at different periods of the progress of development, each of which is of a certain duration.

Partial chorea of individual motor nerves of the organs of sense constitutes some of the most remarkable forms of the disease; such, for instance, as a constant twitching of the eyelids, distortion of the eyes, abnormal motions of the tongue, and almost uninterrupted sneezing. A very common form of partial chorea consists in the constant and unavoidable utterance of inarticulate sounds. As all the motor nerves of the organ of voice are not affected, the patient can still speak, but with effort and stammering, the inarticulate sounds being afterwards made more uninterruptedly and with greater violence. This utterance of sounds frequently continues for weeks, only interrupted during sleep; a long-continued aphonia frequently follows, or a shooting pain on one or the other side of the breast, or asthma. Palpitation of the heart accompanies chorea, both partial and general. In examining individuals suffering from either local or general chorea, it will be very rare not to find tenderness and swelling of some vertebral bone; but such an examination must be repeated, as the sensibility is frequently not discoverable until the disease has lasted for some time. In all the cases of chorea which Dr. Stiebel has examined, he has discovered no other cause than that above mentioned, although he does not deny the possibility of the existence of other causes;—e. g. metastasis of rheumatic inflammation, injuries of the spine, &c. There is doubtless an hereditary disposition to chorea, it having been known to affect whole families. It is particularly frequent among the Jews. Dr. S. has not himself had any opportunity of examining the bodies of individuals who have died of chorea; but, in all the cases which he has read, changes in the spinal marrow, its membranes, or in the spine, are mentioned.

The treatment of chorea is simple. Leeches, followed by mercurial inunctions, and then by more active exutories, are indicated when a painful vertebra is found to exist. If no tender point is at first discoverable, leeches and blisters may be applied along the spine. Calomel is generally given internally as a derivative. If the disease is not thus relieved, repeated cold douche-baths on the spine are almost always of advantage. As a general rule, the disease is cured between the fourteenth and the twenty-first day by this treatment; but, should not this happen, the disease may be left to nature, which almost always put an end to it in the progress of development. At the same time, however, it is necessary to guard against the occurrence of any spinal deviations, and irritant frictions may be applied to the spine. Preparations of iron have sometimes appeared to be of use. The description of the disease which is here given will suffice to account for the credit which such numerous remedies have acquired in the cure of chorea; their use having been coincident with the natural cure. It is not intended to deny that there may be a specific remedy, but only that we at present know not what it is.

*Wochenschrift für die gesammte Heilkunde.* No. I. 1837.

---

*Report on the Inoculation of Morphine, &c. proposed by* Dr. Lafargue. By M. Martin-Solon.

The effects produced by the inoculation of morphine are considered by Dr. Lafargue as worthy of consideration, both in their bearing on practical medicine and on medico-legal questions. If the point of a lancet, dipped in an aqueous solution of morphine, is inserted horizontally, about one line in depth beneath the epidermis, and is allowed to remain there a few seconds, the following effects are observed:—About a minute and a half after the operation, a small pimple, with a diffuse rosy areola, and slightly itching, is observed. In about twenty minutes, the pimple becomes about four lines in diameter, and one line in thickness; it is flattened. Its colour is somewhat more than that of the skin, it is hard, its areola is very red, and about an inch and a half in diameter; its heat has increased, but the sensation of itching remains about the same. During the first hour, the pimple and its areola are at their highest degree of development. From this time, the appearances diminish, and at the end of two or three hours the red colour of the skin has entirely disappeared, the pimple has become very flat; but it does not entirely disappear, until from twelve to twenty-four hours after the operation. If several punctures are made near one another, in the same manner, the appearances of the pimples are as above described, but the areola are confluent; the heat and itching are considerably increased. The appearances, however, disappear in the same time as when a single puncture only has been made. The general effects which Dr. Lafargue experienced from thirteen punctures thus made upon the front of his forearm were, heaviness of the head, frequent yawnings, clamminess of the mouth, and an invincible desire to sleep; the quantity of muriate of morphia employed not having exceeded a quarter of a grain.

The effects just noticed, Dr. L. considers as showing that the inoculation of morphia may supersede the use of blisters and ammoniacal applications, and that it merits employment more particularly where the object of the physician is to produce the local effects of morphine. Its effects as a rubefacient are also very marked. Hence its probable utility in superficial neuralgia and in chronic rheumatism, &c. The local effects produced by the inoculation of belladonna, of strychnine, of sulphate of quinine, were different from those above mentioned. In employing other opiate preparations, such as the laudanum of Sydenham, and solutions of opium in fat, milk, coffee, beer, mucus, acetic acid, and gelatine, the proportion of opium being extremely small, the same results were obtained, and no such effects were produced when any of these substances were inoculated without the opium.

M. Martin-Solon repeated the experiments of Dr. Lafargue. From the inoculation of all the common preparations of opium, he observed the same effects as those above mentioned; except that the papulæ sometimes acquired a diameter of an inch and a half, and that they then became radiated and diffuse. To ascertain whether any other substances were capable of producing the same phenomena, belladonna, strychnine, the gastric juices, chyme, &c., were employed, and the effects which were observed destroyed the exclusiveness which Dr. L. wishes to attribute to the action of preparations of opium.

The conclusion which may be derived from these experiments, may be of some assistance in determining the absence of opium from a fluid which is suspected to contain it; seeing that in all the cases in which fluids containing opium were inoculated (in one instance, the proportion of opium to the solvent was as 1 to 2000,) the phenomena described above were observed by both Dr. Lafargue and M. Solon. The development of the papula can, however, be only regarded as presumptive evidence of the presence of opium; seeing that other substances are capable of producing effects so nearly identical as not to admit of any definite distinction.

Dr. Lafargue has also inoculated a concentrated solution of emetic tartar and the croton oil. The former has always produced a pustule similar to that of acne simplex, containing pus, twenty-four hours after the operation; and the effect of croton oil has constantly been the production of a furuncle thirty-six hours after the introduction of the medicine. Neither of these substances has, however, been sufficiently employed to allow of any inference as to the advantage which this mode of application possesses over that in general use. Its simplicity, nevertheless, renders such an experiment very easy.

*Bulletin de l'Academie Royale de Médecine.* Nos. 1 and 7. 1836-7.

---

*On a peculiar Spasmodic Affection of the Fingers.* By Dr. ALBERS, of Berlin.

DR. ALBERS has met with three cases of this singular affection, in all of which the individual was incapable of holding a pen to write a line. Every attempt to write ended in the pen's slipping away across the paper in an oblique direction; then the thumb, index, and middle finger would begin to tremble, or would move convulsively apart, and the pen finally fall from the hand. In all, the complaint lasted several years without the supervention of other symptoms, and the hand could always be used for any operation except writing, whatever strength or tact it might require. One of these three individuals, though totally unable to use a pen, could easily lift a weight of 100lb. with his right hand. Two of them, with the exception of this complaint, enjoy perfect health, and manifest no other symptom of a nervous affection; a third would seem, from the manner in which he drags his feet in walking, to suffer from disease of the spinal marrow, but he moves without exertion or fatigue, and has been in the same state of health for years. The following is an account of two of the cases:—

CASE I. A bookseller, aged forty-seven, enjoyed the best health till the year 1818, and, indeed, only suffers now from piles and their consequences. It was in the above year that the first symptoms of this complaint manifested themselves. At first it was not of much consequence, and only made it rather more difficult and tedious for him to write. He, therefore, took but little notice of it, particularly as his hand was not weakened in the least, and was quite as useful as before. His medical advisers, to whom he incidentally spoke, attributed the affection to obstinate rheumatism, and recommended liniments accordingly. In a year, however, it had made such rapid progress that it was impossible for him any longer to hold a pen. When he attempted to write, his will had no effect upon the movements of his fingers, and his pen slipped either upwards or downwards, or else his hand began to tremble, whilst the fingers started away from each other, and so let drop the pen. Notwithstanding that it was impossible to write with it, the hand was and is equal to any other exercise, and has never been at all debilitated. Pain has never been felt in it, but in the arm, at first, was a feeling of great fatigue, and in the hand, of cold, as of a current of air. Several physicians held this feeling to be an illusion, but the patient indicates the situation of the superficial volar arch of nerves as its seat, whence, he says, it is continued into the fingers. The remedies which were employed against it, such as arm-baths of hot brandy, frictions with the *acidum formicum*, cold baths, vapour baths, the envelopment of the arm in wax taffety and woollen cloth deeply dyed with indigo, as also animal baths, (which, however, were not long continued,) were totally unavailing. When Dr. A. visited Berlin, for a short time, in 1820, he saw this patient, to whom the ill-success of the treatment to which he had been hitherto subjected was become a source of great anxiety. Excepting his hand, he was quite well, but unable to write, and, therefore, to carry on his business. Dr. A. undertook the case, and, regarding it as purely nervous, he made a small issue half-way between the acromium and the last cervical vertebra; consequently, where the four last cervical nerves and the first dorsal join to form the brachial plexus. He next recommended his patient to make use of a penholder fixed in cork, which, from its larger circumference, was easily held. In three months the issue was productive of the best results. The affection diminished daily; the cork was soon unnecessary; with a common pen, he was able to write as easily and well as he had ever done before, and he believes that the disease would never have returned if the issue had not been closed after a lapse of two years. The consequence of this was, that, six months afterwards, the affection returned in all its former force. A seton was now made where the issue had formerly been, and was kept up for five months, but without avail. The actual cautery, which Dr. V., who was written to, recommended to be applied in two lines from the cervical vertebræ to the tip of the shoulder, the patient's medical attendant did not think it advisable to have recourse to. The patient now determined to learn to write with his left hand: he soon succeeded, and continued in this practice for five years; feeling, it should be remarked, at intervals, a slight attack in this hand, too, of the disease with which the other was permanently affected. About this time his old physician died, and his new one inspired him with fresh hopes of the possibility of a cure. Electricity, galvanism, the baths, &c. of Toeplitz, friction with ammoniacal ointment all over the arm, the application of the moxa in five places from the wrist to the shoulder-joint, sea-bathing, and nux vomica internally, in gradually increased and at last very large doses, were productive of not the slightest result; and the disease is now, after a lapse of twelve years, just what it was at first. The patient has, at last, dismissed all his physicians, and, contented with attempting to remedy the inconvenience of his situation, he uses a wooden apparatus, with which he succeeds in steadying his right hand so as to be able to write with it.

Case II. (communicated by Dr. Siebold, of Gottingen.) This case is of thirty years' standing, and is that of a man of sixty years of age, who has given up all hopes of cure, and who, like the person above mentioned, only seeks to obviate the inconvenience of his situation by mechanical means. To his twenty-seventh year he wrote, with the greatest ease, a perfectly legible hand. His leisure hours he was then accustomed to devote to painting, but this did not at all affect the facility with which he wrote. In 1801, however, when he entered on a situation where he had to write a great deal, he remarked that he could not manage his hand with such ease as before, and that he was obliged to press his thumb more firmly on his pencil and brush. He now gave up painting, for which he found himself incapacitated. Writing, too, began soon to cause him greater difficulty. His thumb moved involuntarily, and pressed without interruption upon the pen, so that his writing became nearly illegible. When he wrote for any length of time, he felt a pain in the first joint of the thumb: in short, the disease rapidly developed itself in the form in which it has maintained to the present. The patient has never been, properly speaking, ill since his childhood, and has never suffered from any eruption. He has always been quite free from gouty and rheumatic pains, and his right hand and arm were originally strong and healthy. He has been subject to violent chronic headaches and to piles, but of late years only. His only great cause of complaint has been the present affection. The four fingers of his right hand are in their normal state; but the thumb, whether he move his hand or not, is continually in motion from right to left. In the first joint there is little action, but the second presses so forcibly towards the palm that, if he hold it with his left hand, and place a pen between his middle and index finger, he cannot write with it, on account of the motion which the pressure of the thumb induces in the latter. Any thing that requires the use of the whole hand he can take hold of, and manage without either difficulty or pain; but he is unequal to any delicate manœuvre where the co-operation of the thumb is required. No intensity of volition can cause the thumb to stay its motion, which it keeps on uninterruptedly. During the twenty years which have elapsed since the first rise of the complaint, numerous physicians have been consulted respecting it. Their prescriptions have been, according as they referred it to gout, paralysis, or nervous weakness, spirituous frictions, tonic arm-baths, moxa, electricity, shower-baths on the hand and arm, &c. &c.; but all have been equally unavailing. In 1808, the patient learnt to write with his left hand, but he exerted it for this purpose to such an extent that he paralysed it, so as to render it nearly useless. Dictation was now his only resource: what he himself wrote was with a thick lead-pencil, which he grasped with his whole hand. Six years ago it was announced in the "General Indicator" that a ring had been found useful in a similar case. On reading this, the patient ordered one to be made like it for himself. It is placed between the first and second joints of the index finger, and encloses with the latter a pen, for holding which, therefore, neither thumb nor middle finger is required. With its aid, he is now able to write tolerably well, notwithstanding that the constant movement of the thumb causes a little uncertainty in all the motions of the hand.

Dr. Albers classes the above affection amongst diseases of the nerves; but whether it be attended with, or independent of, structural derangement, he does not affirm. If we regard merely the impotence of the will to regulate the motions of the fingers, the disease is comparable to St. Vitus' dance, and might be called a *chorea partialis;* but then it generally happens, in these cases, that the fingers take a certain direction, and that the pen, in writing, slips either upwards or downwards.

*Med. Zeitung v. Ver. f. Heilk. in Preussen.* No. 9. 1836.

